# 1,2,3,4-Tetra­hydro­phenazine 5,10-dioxide

**DOI:** 10.1107/S1600536810030242

**Published:** 2010-08-28

**Authors:** Tao Sun, Jianye Li, Hongwei Qiao, Aiyou Hao, Yueming Li

**Affiliations:** a School of Chemistry and Chemical Engineering and Key Laboratory of Colloid and Interface Chemistry of the Ministry of Education, Shandong University, Shanda Nanlu 27, Jinan 250100, People’s Republic of China; b Shandong Shengquan Chemical Co. Ltd, Zhangqiu Jinan, 250204, People’s Republic of China

## Abstract

The complete mol­ecule of the title compound, C_12_H_12_N_2_O_2_, lies on two crystallographic symmetry elements: a twofold axis and a mirror plane. In the mol­ecular structure, the quinoxaline ring and two methyl­ene substituents lie on the mirror plane while the other two methyl­ene groups are disordered about the plane. The crystal packing is stabilized by weak inter­molecular π–π stacking inter­actions with centroid–centroid distances of 3.6803 (7) Å.

## Related literature

For the synthetic preparation, see: Haddadin & Issidorides (1965 ▶); Issidorides & Haddadin (1966 ▶). For background to quinoxaline di-*N*-oxide compounds, see: Edwards *et al.* (1975 ▶) and for their biological activity, see: Urquiola *et al.* (2008 ▶). For a related structure, see: Wang *et al.* (2010 ▶).
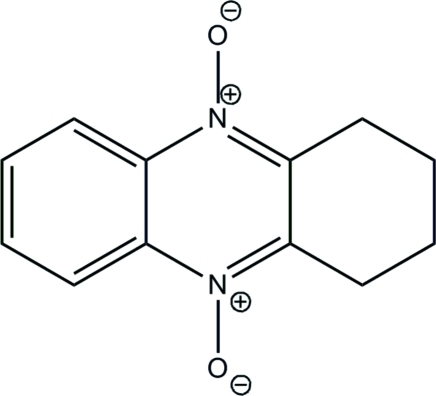


## Experimental

### 

#### Crystal data

C_12_H_12_N_2_O_2_
*M* *_r_* = 216.24
Orthorhombic, 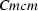

*a* = 11.7780 (2) Å
*b* = 13.1938 (3) Å
*c* = 6.5561 (1) Å
*V* = 1018.80 (3) Å^3^
*Z* = 4
Mo *K*α radiation
μ = 0.10 mm^−1^
*T* = 296 K
0.31 × 0.29 × 0.26 mm

#### Data collection

Bruker APEXII CCD area-detector diffractometer
Absorption correction: multi-scan (*SADABS*; Sheldrick, 1996 ▶) *T*_min_ = 0.67, *T*_max_ = 0.74
3311 measured reflections
620 independent reflections
534 reflections with *I* > 2σ(*I*)
*R*_int_ = 0.016

#### Refinement

*R*[*F* ^2^ > 2σ(*F* ^2^)] = 0.040
*wR*(*F* ^2^) = 0.127
*S* = 1.10
620 reflections
61 parameters
H atoms treated by a mixture of independent and constrained refinement
Δρ_max_ = 0.23 e Å^−3^
Δρ_min_ = −0.29 e Å^−3^

### 

Data collection: *APEX2* (Bruker, 2007 ▶); cell refinement: *SAINT* (Bruker, 2007 ▶); data reduction: *SAINT*; program(s) used to solve structure: *SIR97* (Altomare *et al.*, 1999 ▶); program(s) used to refine structure: *SHELXTL* (Sheldrick, 2008 ▶); molecular graphics: *SHELXTL*; software used to prepare material for publication: *WinGX* (Farrugia, 1999 ▶).

## Supplementary Material

Crystal structure: contains datablocks I, global. DOI: 10.1107/S1600536810030242/nk2042sup1.cif

Structure factors: contains datablocks I. DOI: 10.1107/S1600536810030242/nk2042Isup2.hkl

Additional supplementary materials:  crystallographic information; 3D view; checkCIF report

## Figures and Tables

**Fig. 1.**
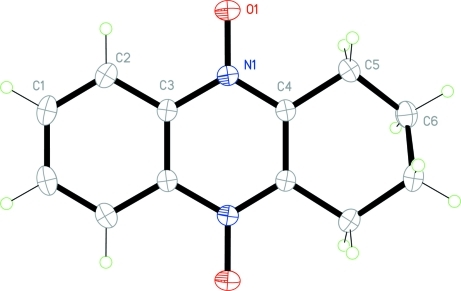
Figure 1. A view of the title compound with displacement ellipsoids are drawn at the 30% probability level. Unlabelled atoms are related to labelled atoms by a twofold rotation. The second disorder component is omitted.

## supplementary crystallographic information

### Comment

Quinoxaline di-N-oxide compounds are widely used in sterilization and growth-promoting of animals, pharmacological properties usable as intermediates for producing plant protection agents (Edwards *et al.*,1975). There has been a growing interest in the syntheses of quinoxaline di-N-oxide compounds that have both biological and commercial importance (Urquiola *et al.,* 2008). Now, we report herein the crystal structure of the title benzotriazole derivative.

The complete molecule of the title compound, C_12_H_12_N_2_O_2_, is generated by a crystallographic symmetry operation along a twofold axis. In the moleclcular structure of the crystal, the quinoxaline ring and two methylene substituents of the quinoxaline ring locate at a mirror plane of the *Cmcm* group. The other two methylenes of the cyclohexane ring are disordered over two positions with half occupancy. The crystal packing is stabilized by weak intermolecular π-π aromatic stacking interactions with centroid-centroid distances of 3.6803 (7) Å.

### Experimental

The compound was synthesized as described previously by Haddadin & Issidorides (1965) and Issidorides & Haddadin (1966). Yellow crystals were obtained by slow evaporation of a methanolic solution.

### Refinement

H atoms in the benzene were placed in geometrically calculated positions and refined using a riding model. H atoms in CH_2_ groups were located in geometrically calculated positions also but their positions were refined independently and their isotropic displacement parameters were fixed to 0.08 in the refinement. Two CH_2_ groups were disordered over symmetry elements and refined with half occupancy.

### Crystal data

**Table:**

C_12_H_12_N_2_O_2_	*F*(000) = 456
*M**_r_* = 216.24	*D*_x_ = 1.410 Mg m^−^^3^
Orthorhombic, *C**m**c**m*	Mo *K*α radiation, λ = 0.71073 Å
Hall symbol: -C 2c 2	Cell parameters from 1577 reflections
*a* = 11.7780 (2) Å	θ = 2.3–26.8°
*b* = 13.1938 (3) Å	µ = 0.10 mm^−^^1^
*c* = 6.5561 (1) Å	*T* = 296 K
*V* = 1018.80 (3) Å^3^	Prism, yellow
*Z* = 4	0.31 × 0.29 × 0.26 mm

### Data collection

**Table:**

Bruker APEXII CCD area-detector diffractometer	620 independent reflections
Radiation source: fine-focus sealed tube	534 reflections with *I* > 2σ(*I*)
graphite	*R*_int_ = 0.016
φ and ω scans	θ_max_ = 26.9°, θ_min_ = 2.3°
Absorption correction: multi-scan (*SADABS*; Sheldrick, 1996)	*h* = −14→14
*T*_min_ = 0.67, *T*_max_ = 0.74	*k* = −16→12
3311 measured reflections	*l* = −8→8

### Refinement

**Table:**

Refinement on *F*^2^	Primary atom site location: structure-invariant direct methods
Least-squares matrix: full	Secondary atom site location: difference Fourier map
*R*[*F*^2^ > 2σ(*F*^2^)] = 0.040	Hydrogen site location: inferred from neighbouring sites
*wR*(*F*^2^) = 0.127	H atoms treated by a mixture of independent and constrained refinement
*S* = 1.10	*w* = 1/[σ^2^(*F*_o_^2^) + (0.0854*P*)^2^ + 0.1004*P*] where *P* = (*F*_o_^2^ + 2*F*_c_^2^)/3
620 reflections	(Δ/σ)_max_ < 0.001
61 parameters	Δρ_max_ = 0.23 e Å^−^^3^
0 restraints	Δρ_min_ = −0.29 e Å^−^^3^

### Special details

**Table:**

Experimental. 1H NMR (400?MHz, DMSO-d6): δ 8.67 (2*H*, d, J = 3.5?Hz, Ar—H), 7.89 (2*H*, d, J = 3.2?Hz, Ar—H), 3.77 (1*H*, s, CH), 2.66 (3*H*, s, CH3), 2.51 (2*H*, m, CH2), 1.45 (6*H*, s, CH3); Calcd for C13H16N2O2: C, 67.22; H, 6.94; N, 12.06. Found: C, 67.18; H, 6.99; N, 11.95; ESIMS calcd for C13H16N2O2H+ m/*z* 232.38, found m/*z* 232.19.
Geometry. All e.s.d.'s (except the e.s.d. in the dihedral angle between two l.s. planes) are estimated using the full covariance matrix. The cell e.s.d.'s are taken into account individually in the estimation of e.s.d.'s in distances, angles and torsion angles; correlations between e.s.d.'s in cell parameters are only used when they are defined by crystal symmetry. An approximate (isotropic) treatment of cell e.s.d.'s is used for estimating e.s.d.'s involving l.s. planes.
Refinement. Refinement of *F*^2^ against ALL reflections. The weighted *R*-factor *wR* and goodness of fit *S* are based on *F*^2^, conventional *R*-factors *R* are based on *F*, with *F* set to zero for negative *F*^2^. The threshold expression of *F*^2^ > σ(*F*^2^) is used only for calculating *R*-factors(gt) *etc*. and is not relevant to the choice of reflections for refinement. *R*-factors based on *F*^2^ are statistically about twice as large as those based on *F*, and *R*- factors based on ALL data will be even larger.

### Fractional atomic coordinates and isotropic or equivalent isotropic displacement parameters (Å^2^)

**Table:**

	*x*	*y*	*z*	*U*_iso_*/*U*_eq_	Occ. (<1)
C1	0.44078 (13)	−0.14330 (10)	0.2500	0.0434 (4)	
H1	0.4017	−0.2045	0.2500	0.052*	
C2	0.38142 (12)	−0.05388 (9)	0.2500	0.0399 (4)	
H2	0.3025	−0.0544	0.2500	0.048*	
C3	0.44062 (11)	0.03818 (9)	0.2500	0.0311 (4)	
C4	0.44068 (10)	0.21722 (9)	0.2500	0.0326 (4)	
C5	0.37292 (12)	0.31337 (10)	0.2500	0.0470 (4)	
H5	0.3247 (10)	0.3113 (9)	0.131 (2)	0.070*	
C6	0.44666 (19)	0.40485 (17)	0.1867 (4)	0.0618 (9)	0.50
H6	0.407 (2)	0.4669 (19)	0.199 (5)	0.090*	0.50
H7	0.468 (3)	0.3993 (19)	0.040 (4)	0.090*	0.50
N1	0.38161 (10)	0.12976 (7)	0.2500	0.0336 (4)	
O1	0.27161 (9)	0.12938 (6)	0.2500	0.0508 (4)	

### Atomic displacement parameters (Å^2^)

**Table:**

	*U*^11^	*U*^22^	*U*^33^	*U*^12^	*U*^13^	*U*^23^
C1	0.0608 (9)	0.0288 (7)	0.0407 (7)	−0.0082 (5)	0.000	0.000
C2	0.0426 (8)	0.0339 (7)	0.0432 (7)	−0.0078 (5)	0.000	0.000
C3	0.0339 (8)	0.0281 (7)	0.0314 (6)	−0.0008 (4)	0.000	0.000
C4	0.0318 (7)	0.0277 (7)	0.0384 (7)	0.0008 (4)	0.000	0.000
C5	0.0372 (8)	0.0320 (8)	0.0719 (10)	0.0056 (5)	0.000	0.000
C6	0.0516 (11)	0.0289 (10)	0.105 (3)	0.0038 (7)	0.0002 (10)	0.0110 (10)
N1	0.0282 (6)	0.0313 (6)	0.0412 (6)	−0.0005 (3)	0.000	0.000
O1	0.0269 (6)	0.0447 (7)	0.0807 (8)	−0.0011 (3)	0.000	0.000

### Geometric parameters (Å, °)

**Table:**

C1—C2	1.3714 (19)	C5—C6^ii^	1.544 (3)
C1—C1^i^	1.395 (3)	C5—C6	1.544 (3)
C1—H1	0.9300	C5—H5	0.965 (13)
C2—C3	1.4005 (17)	C6—C6^ii^	0.831 (5)
C2—H2	0.9300	C6—C6^iii^	1.256 (5)
C3—N1	1.3939 (15)	C6—C6^i^	1.506 (4)
C3—C3^i^	1.399 (2)	C6—H6	0.95 (2)
C4—N1	1.3474 (15)	C6—H7	1.00 (3)
C4—C4^i^	1.397 (2)	N1—O1	1.2956 (16)
C4—C5	1.4987 (16)		
			
C2—C1—C1^i^	120.65 (9)	C6^ii^—C6—C6^iii^	90.000 (2)
C2—C1—H1	119.7	C6^ii^—C6—C6^i^	56.5 (2)
C1^i^—C1—H1	119.7	C6^iii^—C6—C6^i^	33.5 (2)
C1—C2—C3	119.49 (15)	C6^ii^—C6—C5	74.39 (10)
C1—C2—H2	120.3	C6^iii^—C6—C5	124.23 (10)
C3—C2—H2	120.3	C6^i^—C6—C5	108.72 (16)
N1—C3—C3^i^	119.90 (7)	C6^ii^—C6—H6	85.1 (18)
N1—C3—C2	120.24 (14)	C6^iii^—C6—H6	119.6 (16)
C3^i^—C3—C2	119.86 (8)	C6^i^—C6—H6	111.4 (17)
N1—C4—C4^i^	121.08 (7)	C5—C6—H6	112.1 (16)
N1—C4—C5	116.74 (12)	C6^ii^—C6—H7	164.4 (18)
C4^i^—C4—C5	122.17 (7)	C6^iii^—C6—H7	75.0 (18)
C4—C5—C6^ii^	111.23 (14)	C6^i^—C6—H7	108.4 (18)
C4—C5—C6	111.23 (14)	C5—C6—H7	110.3 (16)
C6^ii^—C5—C6	31.2 (2)	H6—C6—H7	106 (2)
C4—C5—H5	106.8 (7)	O1—N1—C4	121.31 (9)
C6^ii^—C5—H5	124.8 (8)	O1—N1—C3	119.68 (9)
C6—C5—H5	97.8 (7)	C4—N1—C3	119.01 (13)
			
C1^i^—C1—C2—C3	0.0	C4—C5—C6—C6^i^	−50.6 (2)
C1—C2—C3—N1	180.0	C6^ii^—C5—C6—C6^i^	45.6 (2)
C1—C2—C3—C3^i^	0.0	C4^i^—C4—N1—O1	180.0
N1—C4—C5—C6^ii^	163.23 (11)	C5—C4—N1—O1	0.0
C4^i^—C4—C5—C6^ii^	−16.77 (11)	C4^i^—C4—N1—C3	0.0
N1—C4—C5—C6	−163.23 (11)	C5—C4—N1—C3	180.0
C4^i^—C4—C5—C6	16.77 (11)	C3^i^—C3—N1—O1	180.0
C4—C5—C6—C6^ii^	−96.23 (6)	C2—C3—N1—O1	0.0
C4—C5—C6—C6^iii^	−17.19 (11)	C3^i^—C3—N1—C4	0.0
C6^ii^—C5—C6—C6^iii^	79.04 (8)	C2—C3—N1—C4	180.0

Symmetry codes: (i) −*x*+1, *y*, −*z*+1/2; (ii) *x*, *y*, −*z*+1/2; (iii) −*x*+1, *y*, *z*.
